# Effect of Ormocer-Based Adhesive and Flowable Composites on Dentine Bond Strength with Snow-Plow Technique

**DOI:** 10.3290/j.ohpd.c_2407

**Published:** 2025-12-12

**Authors:** Cansu Yikici Çöl, Sema Yazici Akbiyik

**Affiliations:** a Cansu Yikici Çöl Specialist in Restorative Dentistry, Panorama Ankara Oral and Dental Health Clinic, Paragon Tower – Kizilirmak District, 1445th Street, No: 2/1, Apt. No: 131, Ankara, 06510, Turkey. Writing – review and editing, writing – original draft, methodology, investigation, and data curation.; b Sema Yazici Akbiyik Assistant Professor, Department of Restorative Dentistry, Faculty of Dentistry, Lokman Hekim University, No: 4, 2179th Street, Söğütözü, 06510 Çankaya, Ankara, Turkey. Writing – review and editing, validation, methodology, formal analysis, data curation, and conceptualisation.

**Keywords:** resin composites, dental adhesive, ormocer, shear bond strength, snow-plow

## Abstract

**Purpose:**

This study investigated the effects of an ormocer-based adhesive and an ormocer-based flowable resin composite on dentine bond strength and failure modes using the snow-plow technique.

**Materials and Methods:**

Sixty-six samples were divided into six subgroups: universal adhesive, ormocer-based adhesive, and their combinations with or without the snow-plow technique (using either nanohybrid or ormocer-based flowable composite). All groups were etched, an adhesive agent was applied, and composite materials were placed. Samp-les were stored at 37°C in distilled water.

**Results:**

No statistically significant differences in bond strength were found among the groups. There was no difference between the universal adhesive and ormocer-based adhe-sive, or between the nanohybrid and ormocer-based flowable composites. The snow-plow technique also did not significantly affect bond strength (p > 0.05).

**Conclusion:**

The use of an ormocer-based flowable composite, an ormocer-based adhesive, or the snow-plow technique did not significantly influence short-term dentine bond strength.

Dental composites are favoured for several reasons, including their natural tooth-like appearance, ease of application, stability, the ability to be polymerised at a convenient time, conservation of tooth structure compared to indirect restorations, repairability and their reasonable cost.^18^ However, dental composites still have several disadvantages. These include polymerisation shrinkage, resin–dentine interface failure, microleakage, cytotoxicity, relatively high coefficient of thermal expansion, and discolouration. In addition, bonding to enamel is effective, stable, and long-lasting,^5^ while bonding to dentine already presents difficulties due to its structure.^21^

The utilisation of composite materials for posterior restorations has significantly increased over recent decades. Nonetheless, the challenge of effectively restoring proximal areas persists. Composites experience volumetric shrinkage of 2–6% during setting, which generates contraction stresses between the composite and the tooth. This strain on the interfacial bond can lead to debonding, microgaps, and cuspal deflection. This phenomenon adversely affects the overall longevity and performance of the restorations.^11,13^

The acronym ‘ormocer’ is the abbreviation for ‘organically modified ceramics’; in the literature also described as ‘ormosils’ (‘organically modified silicates’).^16^ Resin composites consist of an organic matrix and filler particles that provide mechanical and physical properties.^12^ Ormocers are formed by combining nanohybrid technology with organically modified ceramic technology.^17^ In addition to combining the benefits of both organic polymers (such as flexibility and impact resistance) and inorganic materials (such as mechanical strength, chemical resistance, and thermal stability), the larger size of the monomer molecules can also reduce polymerisation shrinkage, wear, and leaching of monomers.^24^

The ‘snow-plow technique’ has been used to compensate for the negative effects of polymerisation stress. In this technique, the first layer of uncured flowable composite is placed, followed by injection and adaptation of a medium-viscosity composite layer. Both layers are then cured simultaneously. This technique was first described by Opdam et al^26^ (2003) and was considered a variation of the sandwich technique, where an intermediate layer of bulk-fill flowable composite was deposited first on the cavity margin, followed by placement of packable composite; both layers were cured simultaneously. It is associated with better clinical performance as the placement of flowable composite allows for better marginal adaptation. Nevertheless, there were few studies concerning the ‘snow-plow’ technique with conflicting opinions.^4,35^ A randomised clinical trial^13^ found that both bulk-fill and snow-plow techniques are highly recommended, with similar clinical outcomes.

For successful results, different restorative materials must exhibit high bond strength values​​. While this may be attributed to the quality of dental adhesives, it has also been observed that the placement techniques of the materials affect the bond strength. There have been limited studies investigating ‘the snow-plow technique’ with conventional flowable composites and adhesive, and data provided a mixed opinion of the efficacy of this placement method.^3,26,29^ Therefore, further investigation is warranted.

Existing studies have mainly investigated the snow-plow technique using conventional nanohybrid composites, and no data are available on the use of ormocer-based flowable composites and adhesives within this protocol. This study aims to investigate the effect of ormocer-based adhesive and ormocer-based flowable resin composite on the shear bond strength to dentine and failure modes when used with the snow-plow technique. The null hypotheses tested were as follows: (1) the use of an ormocer-based adhesive and flowable resin composite does not affect shear bond strength; (2) the application of the snow-plow technique does not affect shear bond strength.

## MATERIALS AND METHODS

Caries-free human molars were collected from The Oral and Maxillofacial Surgery Department Clinic, Faculty of Dentistry, Lokman Hekim University according to the protocol approved by the Scientific Research Ethics Committee of Lokman Hekim University, Ankara, Türkiye (Approval No. 2024/218). Patients were informed about the use of their teeth in the study and provided written informed consent. The brands, batch numbers, types, manufacturers and contents of the materials used in this study are listed in Table 1. Study groups and subgroups, formed by the use of different techniques and materials, are presented in Table 2.

**Table 1:** Materials used in this study

Material type	Polymerization	Contents	Trade name and manufacturers
Universal adhesive	Light (10 s)	Acidic adhesive monomer 10-MDP, Bis-GMA, UDMA, HEMA, ethanol, catalyst, BHT, pyrogenic silicic acid	Futurabond M+ (VOCO, Cuxhaven, Germany)
Ormocer-based, single phase, light-curing adhesive	Light (20 s)	Ormocer resin, dimethacrylates, HEMA, NAF, acid modified methacrylates, CQ, BHT, acetone (1421529)	Admira Bond (VOCO, Cuxhaven, Germany)
Nano-hybrid based flowable restorative material (shade A1)	Light (20 s)	Barium aluminum borosilicate glass, silicon dioxide, HEDMA, BisGMA, TCDDMA, Fumed silica, TEGDMA, BisEMA, initiator, stabilizer, pigment	Grandio SO Light flow (VOCO, Cuxhaven, Germany)
Ormocer-based light-cured flowable restorative material (shade A1)	Light (20 s)	Barium aluminum borosilicate glass, ormocer resin, silicon dioxide, initiator, stabilizer, pigment	Admira fusion flow (VOCO, Cuxhaven, Germany)
Universal, nano-hybrid filling material (shade A1)	Light (20 s)	Barium aluminum borosilicate glass, silicon dioxide, HEDMA, BisGMA, TCDDMA, Fumed silica, TEGDMA, BisEMA, initiator, stabilizer, pigment	Grandio SO (VOCO, Cuxhaven, Germany)


**Table 2:** Study groups

Study groups	Bonding agent	Snow-plow technique	Filling material
Control (Group U)	Universal adhesive	–	Universal, nano-hybrid filling material
Group O	Ormocer-based, single phase, light curing adhesive	–	Universal, nano-hybrid filling material
Group UN	Universal adhesive	Nano-hybrid based restorative material – flowable	Universal, nano-hybrid filling material
Group ON	Ormocer-based, single phase, light curing adhesive	Nano-hybrid based restorative material – flowable	Universal, nano-hybrid filling material
Group UO	Universal adhesive	Ormocer-based restorative material – flowable	Universal, nano-hybrid filling material
Group OO	Ormocer-based, single phase, light curing adhesive	Ormocer-based restorative material –flowable	Universal, nano-hybrid filling material


**Fig 1:**
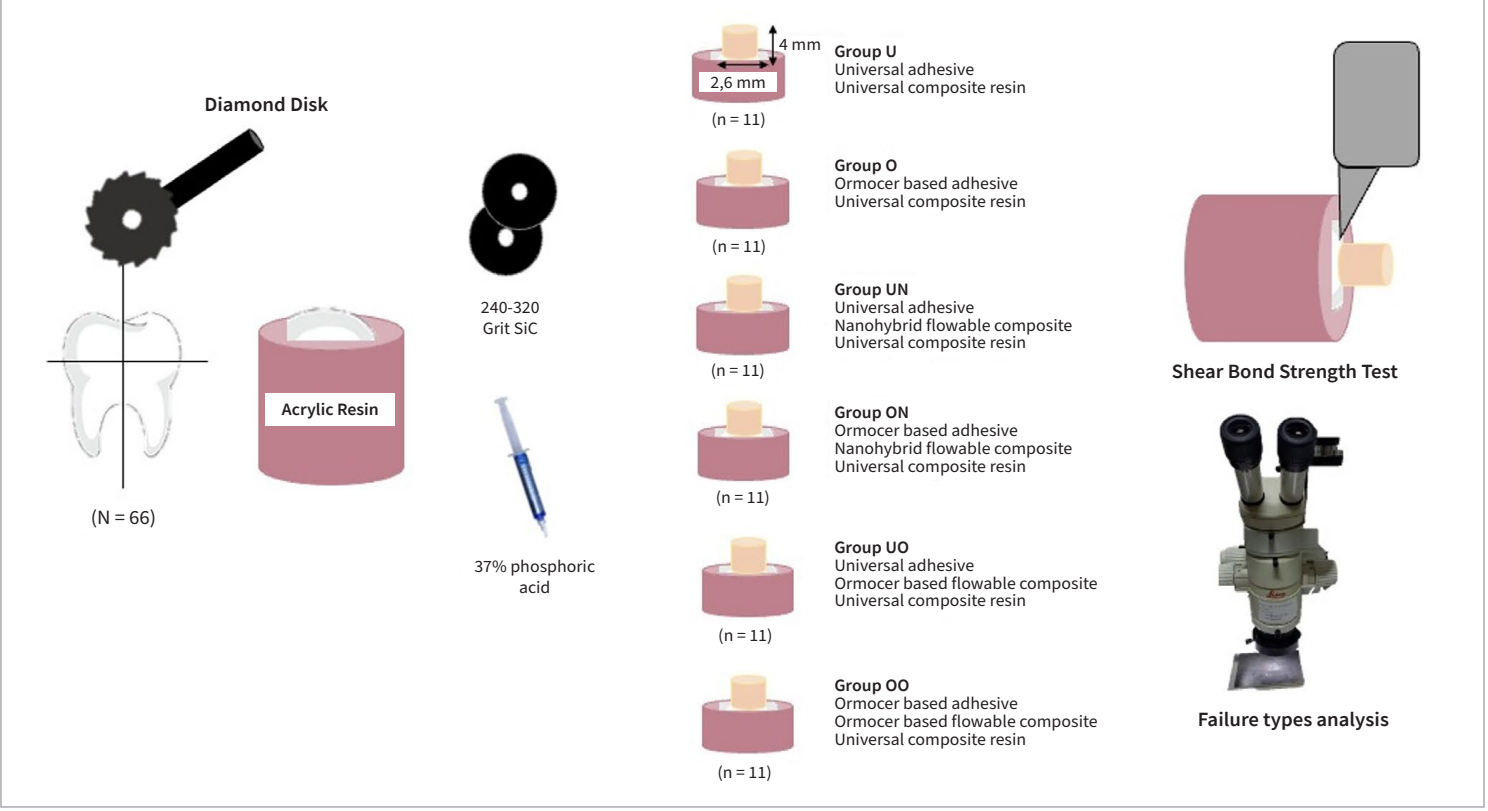
Flowchart of the study.

The overall study design and distribution of samples across groups are summarised in Figure 1 (flowchart). This schematic provides a step-by-step outline of the experimental procedures.

### Sample Preparation

The minimum sample size of the study was determined using G*Power (version 3.1.9.7) with an effect size of 0.50, 90% power, and α = 0.05, resulting in n = 11 specimens per group (total N = 66).

In this *in vitro* study, human molar teeth with extraction indications were visually examined, and those presenting caries, fractures, cracks, restorations, demineralisation, hypomineralisation, or wear were excluded from the study. The selected teeth were cleaned by mechanically removing any deposits, followed by cleaning with pumice and a polishing brush. They were stored in distilled water for a maximum period of 2 months, and the storage water was replaced weekly.

Both the vestibular and palatal/lingual surfaces of the teeth were utilised. The teeth were sectioned at the cementoenamel junction and along the mesiodistal direction using a diamond disc under water cooling. The teeth were embedded in acrylic resin with their buccal or palatal/lingual surfaces facing upward. To protect the specimens from the heat generated during resin polymerisation, they were kept in water during the polymerisation process. In order to obtain a standardised surface area of 4×4 mm^2^, the surfaces were ground using a polishing machine, first with 240 and 320-grit silicon carbide abrasive papers for 20 s. The samples were then stored in deionised water.

The prepared dentine surfaces were etched with 37% phosphoric acid for 15 s according to the manufacturer’s instructions. Following the etching, the surfaces were rinsed with water for 10–20 s and then dried. A total of 66 samples were randomly divided into six groups (n = 11). The study groups are presented in Table 2. Universal adhesive (Futurabond M+, VOCO) was applied to three groups, while ormocer-based adhesive (Admira Bond, VOCO) was applied to the other three groups, and each was polymerised using an LED light device for the time specified by the manufacturer. A plastic tube with dimensions of 2.6 mm × 4 mm (inner diameter × height) was placed on each sample, and the restorative material was applied using the horizontal layering technique, with each layer polymerised for 20 s with an LED curing light. In the groups where the snow-plow technique was used, a flowable resin composite material was placed into the plastic tube and left unpolymerised. Then, the universal nanohybrid filling material (Grandio SO, VOCO) was placed using the horizontal layering technique. The samples were stored at 37°C for 24 h in distilled water before performing the shear bond strength test.

### Shear Bond Strength Test

The shear bond strength test for the prepared test groups was performed using a universal testing machine (Shimadzu IG-IS, Kyoto, Japan) at a speed of 1 mm/min. The obtained Newton values were converted into MPa by calculating the surface area of the samples, and the data were recorded for statistical analysis. The restorative material–dentine interface was examined under a stereomicroscope (Leica MZ 12; Leica Microsystems, Wetzlar, Germany) at 10× magnification, and the types of failures were determined. Failures at the composite/dentine interface were classified as adhesive fractures. Fractures within the composite layer or dentine were classified as cohesive fractures. Adhesive and cohesive fractures occurring together were classified as mixed fractures.^15^

### Statistical Analysis

The data obtained in the study were analysed using SPSS (Statistical Package for the Social Sciences) for Windows 25.0. Descriptive statistical methods (frequency, percentage, minimum–maximum, mean, and standard deviation) were used. The normality of the data was tested using Kolmogorov–Smirnov and Shapiro–Wilk tests. Levene’s test for equality of variances was applied to check the homogeneity of variances between the groups. For normally distributed data, the independent samples t-test was used to compare two groups, while one-way ANOVA was applied for comparisons among more than two groups. Additionally, effect sizes (Cohen’s d and Hedges’ g) with 95% confidence intervals were calculated and interpreted using conventional thresholds (≈0.2 small, ≈0.5 medium, ≈0.8 large). A value of P < 0.05 was considered statistically significant.

## RESULTS

Baseline shear bond strength values and detailed descriptive statistics, including confidence intervals (CI), for all test groups are presented in Table 3.

**Table 3:** Summary of descriptive statistics and confidence intervals by variable

	Mean	Std. Deviation	Interquartile Range (IQR)	95% Confidence Interval for Mean	%5 Trimmed Mean	Min.	Max.
Lower Bound	Upper Bound
**Group U**	27.30	12.15	20.80	19.14	35.47	27.01	11.14	48.71
**Group UN**	26.60	10.46	18.21	19.58	33.63	27.12	**6.31**	37.52
**Group UO**	**26.39**	12.01	**10.54**	**18.32**	34.46	**25.83**	11.84	51.05
**Group O**	**32.22**	**9.48**	16.53	25.85	38.60	**32.33**	18.85	43.74
**Group ON**	31.45	**16.33**	**29.90**	20.47	**42.42**	31.51	7.61	**54.18**
**Group OO**	29.88	10.94	18.85	22.52	37.23	29.88	11.39	48.36
(Group U: Universal adhesive, O: Ormocer-based adhesive, UN: Universal adhesive + Nano-hybrid flowable, ON: Ormocer-based adhesive + Nano-hybrid flowable, UO: Universal adhesive + Ormocer-based flowable, OO: O: Ormocer-based adhesive + Ormocer-based flowable).

The Kolmogorov–Smirnov and Shapiro–Wilk tests indicated that the assumption of normality was met for all groups except Group UO. In this group, the Kolmogorov–Smirnov test revealed a significant deviation from normality (P = 0.027), whereas the Shapiro–Wilk test result was not significant (P = 0.062). All other groups showed P values greater than 0.05 in both tests, confirming normal distribution. Levene’s test confirmed the homogeneity of variances (P >0.05), and the independent samples t-test revealed no significant differences between the groups (P >0.05). ANOVA also showed no statistically significant differences in shear bond strength among the groups (F = 0.490, P = 0.783). The Bonferroni-adjusted post-hoc analysis revealed no statistically significant differences in mean values between any of the groups (all P >0.05, Bonferroni P = 1.000). All pairwise comparisons yielded confidence intervals that included zero, confirming the absence of significant differences.

In this study, the groups with ormocer-based adhesive (31.18 ± 12.24) demonstrated higher shear bond strength values than those with universal adhesives (26.77 ± 11.20); however, this difference was not statistically significant (P >0.05, Cohen’s d = 0.38, small effect size ) (Table 4).

**Table 4:** Universal adhesive and ormocer-based adhesive

	Min	Max	Mean	SD	t-test	P
**Universal adhesive**	6,3197	51,0551	26,7708	11,2083	–1,529	0,131
**Ormocer-based adhesive**	7,6135	54,1898	31,1879	12,2443
(Universal adhesive: Group U + UN + UO, Ormocer-based adhesive: Group O + ON + OO).

When the effectiveness of the snow-plow technique was evaluated, Group U (27.30 ± 12.15) produced higher shear bond values than Group UN (26.60 ± 10.46) and Group UO (26.39 ± 12.01), but with a negligible effect size (Group U and Group UN: Hedges’ g = 0.05, Group U & Group UO: Hedges’ g = 0.07). Group O (32.22 ± 9.48) produced higher shear bond strength values than Groups ON (31.45 ± 16.33) and Group OO (29.88 ± 10.94). A negligible effect size was observed between Groups O and ON (Hedges’ g = 0.05), while a small effect size was observed between Groups O and OO (Hedges’ g = 0.22, small effect size).

The use of a universal adhesive (Group U) and an ormocer-based adhesive (Group O) resulted in no statistically significant difference in shear bond strength values (P >0.05). However, the effect size was medium (Hedges’ g = –0.4, small-medium effect size). This result shows that the mean of Group O was higher than Group U, but the difference did not reach statistical significance due to the small sample size. However, all ormocer-based adhesive groups (Groups O, ON, OO) exhibited higher shear bond strength values than the universal adhesive groups (Groups U, UN, UO) with medium and small effect size. (Groups O & UN: Hedges’ g = 0.54: medium, O & UO: Hedges’ g = 0.51: medium, ON & U: Hedges’ g = 0.27: small, OO & U: Hedges’ g = 0.21, small, ON & UN: Hedges’ g = 0.33: small, ON & UO: Hedges’ g = 0.33: small, OO & UN: Hedges’ g = 0.29: small, OO & UO: Hedges’ g = 0.29: small). Similarly, when the snow-plow technique was applied with different adhesives, no significant difference was observed between the universal adhesive (Groups UN, ON, Hedges’ g = –0.33, small effect size) and ormocer-based adhesive (Groups UO, OO, Hedges’ g = –0.29, small effect size) (P >0.05).

Evaluating the effect of different flowable composites used in the snow-plow technique revealed no statistically significant differences in shear bond strength between nanohybrid and ormocer-based flowable materials within either the universal adhesive groups (Groups UN, UO, Hedges’ g = 0.01, negligible) or the ormocer-based adhesive groups (Groups ON, OO Hedges’ g = 0.10, negligible).

**Fig 2:**
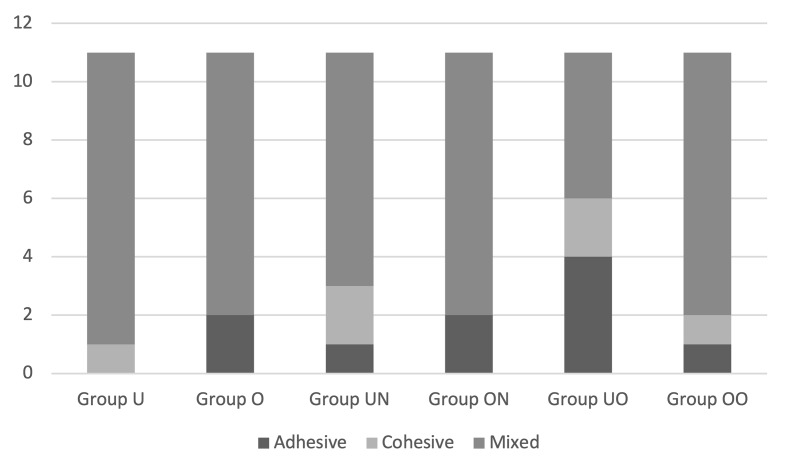
Representative images of failure types.

Failure mode analysis revealed that mixed failures were the most frequent across all groups, followed by cohesive and adhesive failures. Notably, Group U showed no adhesive failures, while Group UN and Group UO presented a higher proportion of cohesive failures. In contrast, Group UO demonstrated the highest incidence of adhesive failures compared with other groups. These distributions are illustrated in Figure 2, which summarises the relative frequency of adhesive, cohesive, and mixed failures across all groups.

## DISCUSSION

Studies over the past decade have yielded mixed findings regarding the effectiveness of ormocer-based systems in enhancing bonding performance. In this study, it is to evaluate the effect of using an ormocer-based adhesive and a flowable composite in combination with the snow-plow technique on shear bond strength, and it makes a unique contribution in this respect. Based on the findings, neither the adhesive system, the resin composite type, nor the application technique significantly affected the shear bond strength results; therefore, the null hypotheses tested in this study can be accepted.

The selection of an appropriate bond strength test represents a critical methodological aspect. In this study, the shear bond strength method was used. Although the microtensile bond strength (µTBS) test offers greater sensitivity by providing more uniform stress distribution and detecting subtle interfacial differences,^31^ it requires labour-intensive preparation and is prone to pre-testing failures due to the small specimen size (≈1 mm^2^). Prior research has noted that µTBS reduces cohesive fractures, whereas shear bond strength is easier to standardise and less time-consuming, particularly when tooth availability is limited.^10^ Since our aim was to compare the effects of adhesives, composite types, and snow-plow technique on short-term dentine bond strength, shear bond strength was preferred to minimise specimen loss and ensure reproducibility. Nonetheless, we acknowledge that µTBS could provide additional insights into interfacial integrity and long-term stability.

Adhesives and composite materials are developing rapidly. However, polymerisation shrinkage remains a significant challenge. Modern composites exhibit a shrinkage of approximately 1–3% during polymerisation. Adhesive systems designed to provide strong bonds exceeding 20 MPa may not always withstand shrinkage stresses, which typically range from 13 to 17 MPa. Consequently, separations and cracks may occur.^11^ This issue is particularly prominent in the gingival region, where the absence of an enamel margin can lead to complications such as poor marginal adaptation, microleakage, secondary caries, postoperative sensitivity, marginal discolouration, cuspal deflection, and even restoration failure. Addressing these challenges is critical to ensuring the long-term success and reliability of dental restorations.^4,23^

Ormocer achieved higher microtensile bond strength and less microleakage than a conventional composite, supporting the idea that ormocer chemistry can form a reliable hybrid layer.^7,32^ This may be attributed to the multifunctional silane network within the ormocer structure, which could enhance infiltration and stability at the adhesive interface.^34^

The comparison between conventional composites and ormocer-based materials revealed similar clinical outcomes in terms of microleakage^6^ and marginal integrity scores.^28^ Furthermore, after 18 months, an ormocer-based monochromatic composite demonstrated a 93% retention rate in caries-free cervical lesions.^22^ In the present study, no statistically significant difference was observed between the ormocer and nanohybrid composite groups (P >0.05 )with a negligible effect size, which is consistent with the findings of a previous systematic review and meta-analysis.^24^ Any potential subtle advantages, such as improved bonding to caries-affected dentine or reduced degradation at the adhesive interface, require further investigation.^33^ Overall, current evidence supports that ormocer systems can achieve reliable shear bond strengths comparable to traditional resin-based materials, provided that optimal technique and adhesive strategies are employed. However, claims of dramatically superior adhesion remain unsubstantiated in most clinical scenarios.^6^

The 1-, 2-, 3-, and 4-year overall survival rates of posterior composite restorations placed using the snow-plow technique were 99%, 96.2%, 89.6%, and 79%, respectively, and were similar to those achieved with the conventional technique. This retrospective study demonstrated that the snow-plow technique did not improve the clinical survival of posterior composite restorations.^3^ Additionally, no difference in microleakage was observed between the conventional and snow-plow technique.^27^ In a limited number of bond strength studies on the snow-plow technique, it has been shown that this approach can yield similar results to the incremental layering technique, regardless of artificial ageing or application time.^25,35^ The findings of the present study are consistent with these previous results. The results of this study indicate that the type of restorative composite and the application method had a negligible or small effect on the short-term shear bond strength of the restorations. However, one of the limitations of this study was that it did not include the assessment of larger sample sizes or various ageing protocols, which may affect long-term outcomes.

In a study evaluating different bonding protocols on ormocer discs, universal adhesives (13.9 ± 3.4 MPa) and a silane and repair composite combination (16.3 ± 2.9 MPa) showed significantly greater immediate repair strength than the ormocer-based adhesive (10.8 ± 2.4 MPa).^9^ Another study reported that the ormocer-based adhesive exhibited the highest shear bond strength to dentine (21.79 MPa) among all adhesives tested, including universal adhesives.^14^ Additionally, a study comparing bulk-fill composite, ormocer, Alkasit, and a conventional composite on caries-affected dentine in primary teeth found that ormocer exhibited the highest µTBS values, while the conventional composite demonstrated the lowest.^33^ Although the ormocer-based adhesive groups showed higher shear bond strength than the universal adhesives, both with and without the snow-plow technique.

In the present study, no statistically significant differences were detected; however, effect size calculations indicated small to medium effects in favour of the ormocer-based groups. This suggests possible trends that could be clinically relevant. Regarding statistical power, our a priori power analysis was designed to detect medium effect sizes with 90% power (α = 0.05). Therefore, while the study was adequately powered for moderate differences, it may not have been sensitive enough to capture smaller but potentially meaningful effects. This result provides a strong recommendation for increasing sample sizes to confirm and expand the findings.

The most notable advantages of ormocer-based composites are their lower polymerisation shrinkage and shrinkage stress, which enhance marginal adaptation and reduce stress at bonded interfaces.^2^ Moreover, due to their pre-polymerised structure and the absence of Bis-GMA, these materials release less residual monomer and exhibit improved biocompatibility.^32^ A cell culture study^8^ has shown that ormocer composites are less cytotoxic to pulp stem cells and may promote the expression of healing markers. The absence of Bisphenol-A derivatives represents another important advantage from a patient safety perspective.^19^ However, despite these benefits, ormocer materials are associated with higher water sorption, which may accelerate the hydrolytic degradation of their mechanical properties and contribute to increased marginal wear or microcrack formation over time. To date, the clinical longevity of ormocer restorations has not yet demonstrated superiority to conventional composites.^1,19^ Nevertheless, the handling and curing requirements of ormocer-based materials remain comparable to those of traditional resin composites.

Cohesive failures typically indicate that the hybrid layer remains structurally stable, whereas adhesive failures suggest debonding at the adhesive interface, as observed under microscopic analysis. Cohesive fractures within the luting agent or restorative material are generally interpreted as indicators of reliable internal strength and bonding. In contrast, adhesive failures are often associated with insufficient bond strength and are more frequently observed in mixed or purely adhesive failure patterns.^30^ Group U exhibited no adhesive failures, whereas Group UO demonstrated the highest incidence of adhesive failure. All tested materials presented micro-shear bond strength (µSBS) values exceeding the clinically acceptable threshold (10–13 MPa),^20^ indicating their suitability for clinical application.

Several limitations should be acknowledged in this study. Observed differences between adhesives may result from variables such as the type of restorative material, surface preparation methods, and testing protocols. Therefore, further research is warranted to identify the most suitable bonding systems for diverse clinical scenarios. Both *in vitro* and *in vivo* studies involving larger sample sizes and long-term ageing protocols are necessary to evaluate the performance of different adhesive strategies more comprehensively. In particular, the lack of ageing protocols or long-term performance data (eg, thermocycling, extended water storage) represents an important limitation of the present investigation, as short-term testing may not fully reflect clinical durability. Moreover, although SBS testing allowed reproducible and standardised specimen preparation, µTBS testing could provide additional sensitivity and more detailed insights into the adhesive interface. Future studies incorporating µTBS together with clinically relevant ageing procedures are therefore recommended to better assess long-term outcomes.

Within the limitations of this study:

1. The ormocer-based adhesive exhibited higher shear bond strength values compared to the universal adhesive, although the difference was not statistically significant.
2. The use of resin composite materials in combination with the snow-plow technique did not contribute to an increase in dentine shear bond strength.
3. There was no significant difference in dentine shear bond strength between the use of an ormocer-based flowable composite and a nanohybrid-based flowable composite when applied with the snow-plow technique.

### Acknowledgements

The authors thank VOCO Dental Products for donating the materials used in this study.

#### Clinical trial

This study is a laboratory-based investigation and does not involve a clinical trial. Therefore, no clinical trial number has been assigned.

#### Ethics approval and consent to participate declarations

This study was conducted with the approval of the ethics committee number 2024/218 from the Scientific Research Ethics Committee of Lokman Hekim University.

The molars gathered were extracted for therapeutic purposes not connected to the study and with prior informed consent from healthy individuals receiving dental care at The Oral and Maxillofacial Surgery Department Clinic, Faculty of Dentistry, Lokman Hekim University. The patients willingly donated the extracted teeth to the Faculty for research purposes.
